# CD4+ T-Cell Count at Antiretroviral Therapy Initiation in the “Treat-All” Era in Rural South Africa: An Interrupted Time Series Analysis

**DOI:** 10.1093/cid/ciab650

**Published:** 2021-07-26

**Authors:** H Manisha Yapa, Hae-Young Kim, Kathy Petoumenos, Frank A Post, Awachana Jiamsakul, Jan-Walter De Neve, Frank Tanser, Collins Iwuji, Kathy Baisley, Maryam Shahmanesh, Deenan Pillay, Mark J Siedner, Till Bärnighausen, Jacob Bor

**Affiliations:** 1 The Kirby Institute, University of New South WalesSydney, NSW, Australia; 2 Africa Health Research Institute, KwaZulu-Natal, South Africa; 3 New York University Grossman School of Medicine, New York, New York, USA; 4 King’s College Hospital National Health Service Foundation Trust, London, United Kingdom; 5 Heidelberg Institute of Global Health, Faculty of Medicine and University Hospital, University of Heidelberg, Heidelberg, Germany; 6 College of Health Sciences, University of KwaZulu-Natal, Durban, South Africa; 7 Lincoln International Institute for Rural Health, University of Lincoln, Lincoln, United Kingdom; 8 Centre for the AIDS Programme of Research in South Africa, University of KwaZulu-Natal, Durban, South Africa; 9 Department of Global Health and Infection, Brighton and Sussex Medical School, Brighton, United Kingdom; 10 Faculty of Epidemiology and Population Health, London School of Hygiene & Tropical Medicine, London, United Kingdom; 11 Institute for Global Health, University College London, London, United Kingdom; 12 Division of Infection & Immunity, University College London, London, United Kingdom; 13 Massachusetts General Hospital and Harvard Medical School, Boston, Massachusetts, USA; 14 Harvard T.H. Chan School of Public Health, Boston, Massachusetts, USA; 15 Department of Global Health and Epidemiology, Boston University, Boston, Massachusetts, USA; 16 Health Economics and Epidemiology Research Office, Wits Health Consortium, Faculty of Health Science, University of Witswatersrand, Johannesburg, Gauteng, South Africa

**Keywords:** HIV/AIDS, universal test and treat (UTT), CD4, antiretroviral therapy initiation

## Abstract

**Background:**

South Africa implemented universal test and treat (UTT) in September 2016 in an effort to encourage earlier initiation of antiretroviral therapy (ART).

**Methods:**

We therefore conducted an interrupted time series (ITS) analysis to assess the impact of UTT on mean CD4 count at ART initiation among adults aged ≥16 years attending 17 public sector primary care clinics in rural South Africa, between July 2014 and March 2019.

**Results:**

Among 20 599 individuals (69% women), CD4 counts were available for 74%. Mean CD4 at ART initiation increased from 317.1 cells/μL (95% confidence interval [CI], 308.6 to 325.6) 1 to 8 months prior to UTT to 421.0 cells/μL (95% CI, 413.0 to 429.0) 1 to 12 months after UTT, including an immediate increase of 124.2 cells/μL (95% CI, 102.2 to 146.1). However, mean CD4 count subsequently fell to 389.5 cells/μL (95% CI, 381.8 to 397.1) 13 to 30 months after UTT but remained above pre-UTT levels. Men initiated ART at lower CD4 counts than women (–118.2 cells/μL, 95% CI, –125.5 to –111.0) throughout the study.

**Conclusions:**

Although UTT led to an immediate increase in CD4 count at ART initiation in this rural community, the long-term effects were modest. More efforts are needed to increase initiation of ART early in those living with human immunodeficiency virus, particularly men.

## Teaser

Universal test and treat (UTT) elicited an immediate increase in CD4 count at antiretroviral therapy (ART) initiation in rural South Africa, with modest long-term effects. Men initiated ART at lower CD4 counts than women. UTT must be combined with other interventions to sustain early ART initiation.

**(See the Editorial Commentary by Ford and Chiller on pages 1360–1.)**

Universal test and treat (UTT) aims to rapidly reduce AIDS-related deaths and incident human immunodeficiency virus (HIV) infections [1]. CD4+ T-cell counts are no longer required to determine antiretroviral therapy (ART) eligibility among people living with HIV [2, 3]. However, there are strong clinical indications to continue baseline CD4 testing, even with rapid ART initiation (ART initiation within 7 days of HIV diagnosis) [4], including that the CD4 count provides critical information on immune status and risk of opportunistic infections, enabling timely clinical interventions [2, 3] and disease care packages via a public health approach [4] as clinical stage does not accurately reflect actual immune status [5]; many individuals in low- and middle-income countries (LMIC) still present late to care [6, 7]; many individuals who reengage with care after treatment interruption have advanced HIV [8]; and risk of mortality is highest during the first few months of ART among those with lower CD4 counts at initiation [9, 10].

To initiate ART early in HIV infection, people living with HIV must first know their HIV status, link to care, and initiate ART. One summary measure of the timing of ART initiation in relation to HIV seroconversion is CD4 count at ART initiation. Unlike previous requirements for regular CD4 measurements until ART eligible, under UTT we expect most individuals to have a single CD4 measurement, their baseline CD4 measurement, which serves the dual function of CD4 count at diagnosis and CD4 at ART initiation. Two previous studies in sub-Saharan Africa showed mixed results on the impact of CD4 eligibility expansions (to <350 cells/μL) on CD4 count at ART initiation [6, 11]. Moreover globally, while median CD4 counts at ART initiation increased between 2002 and 2015, they remained <350 cells/μL and increases were larger among women than among men in LMIC [12].

In January 2015, South Africa removed CD4 eligibility criteria for pregnant or breastfeeding women (Option B+) and expanded CD4 eligibility criteria to ≤500 cells/μL for nonpregnant or breastfeeding adults [13]. South Africa implemented UTT in September 2016 [14]. A critical question is whether this ambitious policy change resulted in ART initiation earlier in HIV infection as anticipated.

We hypothesized that CD4 count at ART initiation would increase rapidly after UTT implementation as the backlog of people living with HIV who previously presented with CD4 counts >500 cells/μL became eligible for ART. Following this short-term increase, we hypothesized that CD4 counts at ART initiation would remain high and continue increasing. We therefore examined short- and medium-term effects of UTT on mean CD4 count at ART initiation among men and women attending public sector primary care clinics in rural South Africa.

## METHODS

### Study Setting and Design

This longitudinal study was conducted in the Hlabisa subdistrict of northern KwaZulu-Natal, at 17 nurse-led primary care clinics that are overseen by the local district hospital. CD4 and HIV viral load monitoring are routinely available. HIV prevalence is 30% [15]. Routine clinical data from the South African national ART program (TIER.Net) were sourced via the Africa Health Research Institute (AHRI) which has an agreement with the Department of Health (DoH) to access routine clinical data for research purposes. National ART program data are captured by DoH clerical staff from patient medical records onto the TIER.Net database.

AHRI has operated a longitudinal population health and demographic surveillance system (HDSS) since 2003 in the area [16]. Since 2017, the AHRI HDSS has offered home-based HIV testing and linkage-to-care support and facilitates ART initiation at DoH clinics [17]. Additional information on the study setting is provided in the Supplementary Material.

During all time periods analyzed in the present study, South African ART guidelines recommended baseline CD4 counts. Prior to 2015, CD4 testing was required at diagnosis for all adults and then every 6 months until ART eligible [18]. Baseline CD4 testing was also required during the Option B+ era [13]. Even during UTT (September 2016 onward) and the move toward ART initiation on the same day as HIV diagnosis, the CD4 count has remained a recommended baseline investigation [19].

### Participants

We included all women and men living with HIV aged ≥16 years who commenced ART between 1 July 2014 and 31 March 2019 at the 17 primary care clinics in Hlabisa subdistrict.

### Outcomes and Exposures

The outcome of interest was CD4 count at ART initiation. We defined this as the closest CD4 count to the ART initiation date within a window of up to 6 months prior to the ART start date, or up to 3 months after the ART start date.

The main exposure of interest was calendar time by periods of CD4 count-based ART eligibility according to South African guidelines (Option B+ guideline in January 2015 and UTT guideline in September 2016) and by allowing 12 months for each new guideline to assimilate. We therefore divided calendar time as follows: prior to 1 January 2015 (“pre-Option B+”); 1 January 2015 to 31 December 2015 (the 12 months following the Option B+ policy, titled “option B+ implementation” which occurred 9–20 months prior to UTT); 1 January 2016 to 31 August 2016 (“pre-UTT” which was 1–8 months prior to UTT); 1 September 2016 to 31 August 2017 (“UTT implementation” which was 1–12 months following UTT); and 1 September 2017 to 31 March 2019 (“post UTT” which was 13–30 months following UTT).

### Data Sources

We analyzed data from the South African national ART clinical database, TIER.Net, which registers and follows all individuals living with HIV from ART initiation [20]. TIER.Net includes laboratory results, ART regimen, ART switches, and visit dates. Pregnancy status was not available.

### Statistical Analyses

We first summarized CD4 count categories at ART initiation by sex and time period. We also described time trends in the proportion of individuals initiating ART without a baseline CD4 count, due to concerns that health workers may not adhere to guideline recommendations. We then graphically represented time trends in mean CD4 count at ART initiation by sex using flexible semiparametric regression methods, namely, kernel-weighted smoothed polynomial regression [21].

To measure the impact of UTT on CD4 count at ART initiation, we performed segmented linear regression (interrupted time series [ITS] analyses) with a continuous time variable, binary exposure variables for each policy change, and time-by-policy interaction terms [22]. The time-by-policy interaction term reflects the difference in slope between consecutive time periods (trend change) [22]. As each policy change may have taken time to assimilate, we allowed additional trend changes 12 months after each official policy. We adjusted for missing CD4 counts by inverse probability weighting [23]. Specifically, we regressed availability of a within-window CD4 count on age category and clinic attended for ART initiation. We then used the inverse of the predicted probability of having a CD4 count within the specified window in our main ITS regression model. We selected inverse probability weights over imputation methods to avoid making assumptions about unobserved reasons for missingness.

We also adjusted for sex in our main ITS regression model. Using regression post-estimation commands, we estimated average CD4 counts and actual trends (slopes) for each time period. We performed all statistical analysis in Stata 15.0 (StataCorp, 2017, College Station, TX).

### Ethical Approvals

The University of KwaZulu-Natal Biomedical Research Ethics Committee and the University of New South Wales Human Research Ethics Committee provided ethical approval.

## RESULTS

A total of 20 599 individuals (69% women) aged ≥16 years commenced ART between July 2014 and March 2019. This included 10 993 ART initiators after the UTT policy change in September 2016. Median age at ART initiation was 30 years (interquartile range, 25–38). The ART regimen at initiation was tenofovir disoproxil fumarate, emtricitabine, and efavirenz in 98% individuals.

**Figure 1.**
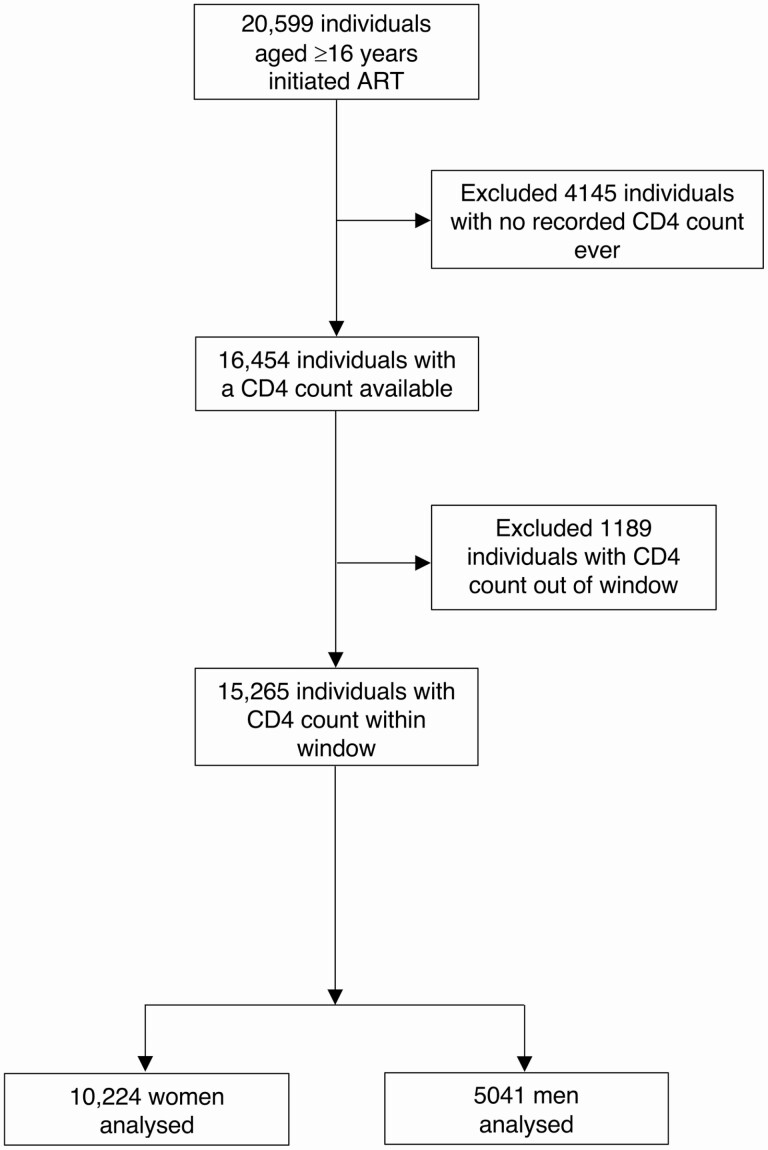
Participant flow diagram for inclusion in regression models. Abbreviation: ART, antiretroviral therapy.

**Figure 2.**
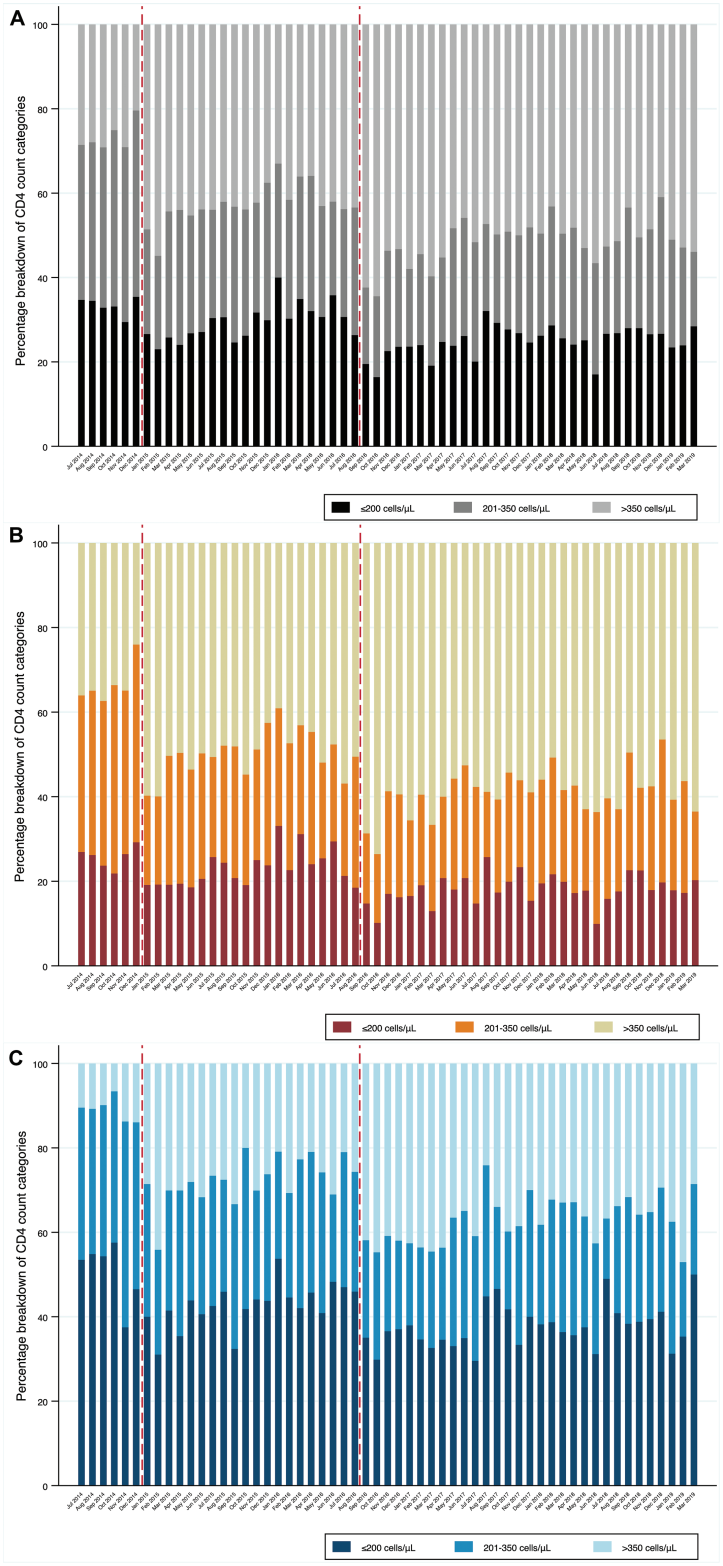
CD4 categories overall *(A)* and by sex,women *(B)* and men *(C)*.

A CD4 count was available for 16 454 (80%) ART initiators, of whom 15 265 had a CD4 count within the specified window (Figure 1). Most (97%) CD4 counts within the window were on or prior to the ART start date. Among the 15 265 individuals with a within-window CD4 count, 67% were women (Figure 1). Men initiating ART were older and had lower CD4 counts than women (Figure 2, Supplementary Table 1).

A large proportion of individuals initiated ART at CD4 counts ≤200 cells/μL even after UTT. Compared with Option B+ implementation and pre-UTT periods, there were slight reductions in the proportion of individuals initiating ART at CD4 counts ≤200 cells/μL after UTT (Figure 2, Supplementary Table 1). The total proportion of individuals initiating ART at CD4 counts ≤200 cells/μL ranged from 27%–32% prior to the UTT policy change to 22%–26% after the UTT policy change.

**Figure 3.**
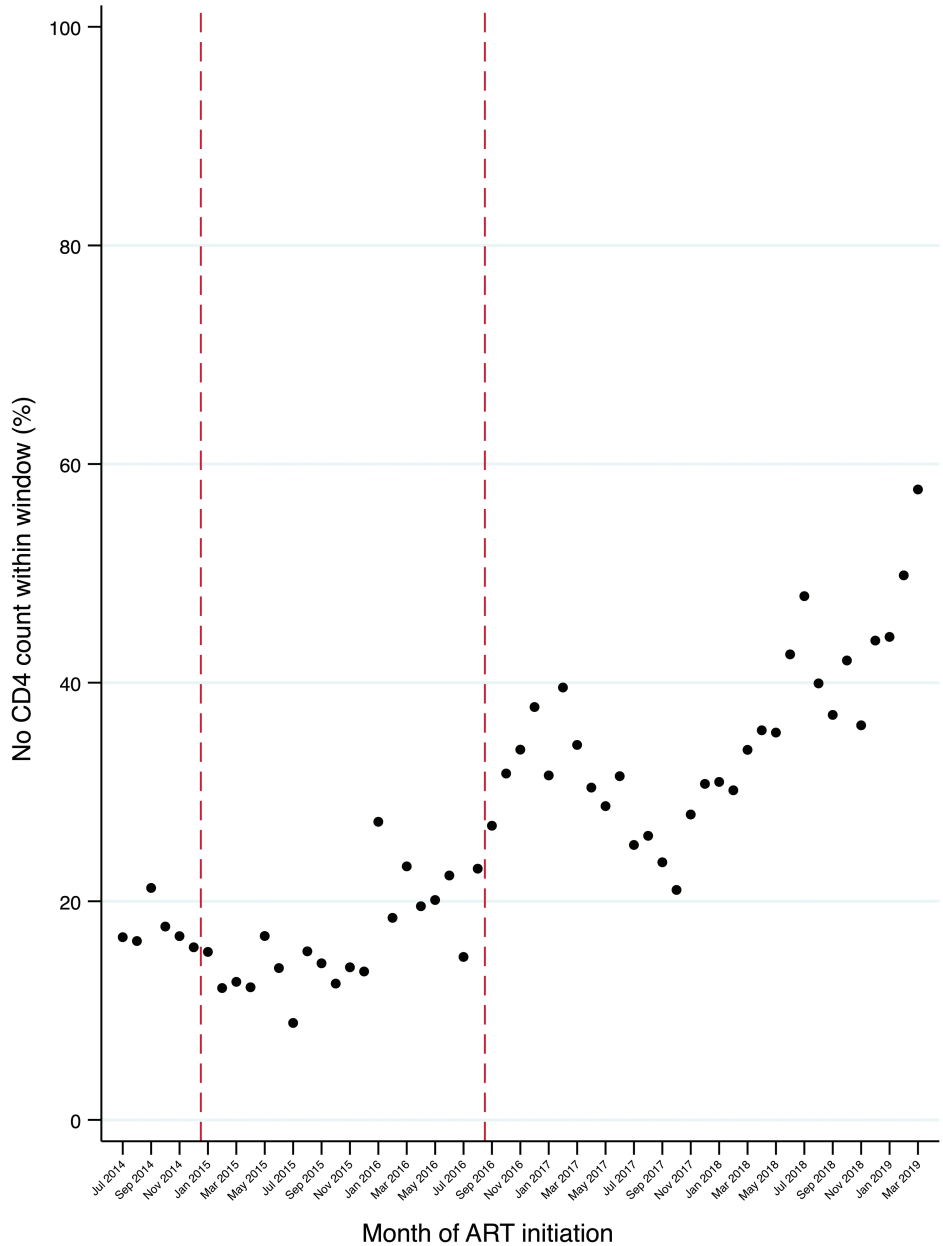
Time trends in proportion of individuals without a within-window CD4 count recorded. Dashed vertical lines depict policy change: January 2015, CD4 eligibility cutoff ≤500 cells/μL for adults or Option B+ for pregnant/breastfeeding women (Option B+ era); September 2016, Universal Test and Treat. Abbreviation: ART, antiretroviral therapy.

The proportion of ART initiators without a baseline CD4 count increased over time. Among the 5334 individuals without a CD4 count within the specified window, 4145 (78%) did not have a CD4 performed at all (Figure 1). The proportion of individuals who initiated ART without a CD4 count appeared to increase with larger clinic size and was greater among women (Supplementary Table 2). There was an increasing time trend in the proportion of all individuals who initiated ART without a within-window CD4 count, particularly after September 2016 (Figure 3).

**Figure 4.**
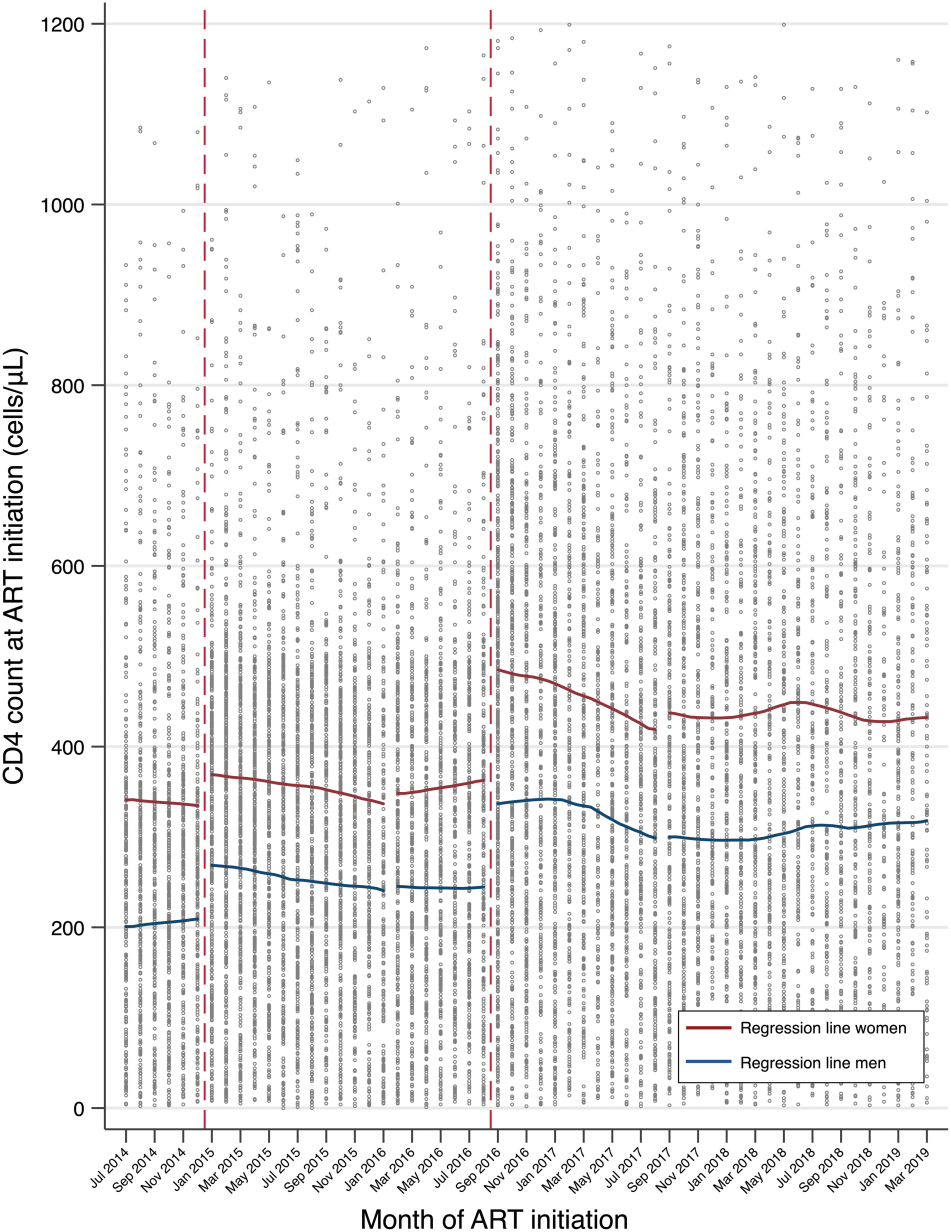
CD4 count at ART initiation among women and men. Dashed vertical lines depict policy change: January 2015, CD4 eligibility cutoff ≤500 cells/μL for adults or Option B+ for pregnant/breastfeeding women (Option B+ era); September 2016, Universal Test and Treat. Abbreviation: ART, antiretroviral therapy.

Time trends in CD4 count at ART initiation are depicted in Figure 4. Women consistently initiated ART at higher CD4 counts than men. There was a marked increase in CD4 count at ART initiation immediately after UTT, followed by a downward trend and stabilization thereafter.

### Impact of Policy Changes on CD4 Count at ART Initiation

#### January 2015 to August 2016 (Option B+ implementation and pre-UTT periods)

Immediately following the policy change in January 2015, mean CD4 count at ART initiation increased by 52.7 cells/μL (95% confidence interval [CI], 30.9 to 74.5). There was an upward absolute trend in CD4 count at ART initiation during pre-UTT of 3.1 cells/μL per month (95% CI, 0.5 to 5.7; Table 1).

**Table 1.** Interrupted Time Series Regression: Mean CD4 Count at Antiretroviral Therapy Initiation by Time Period

Model parameter	Pre-Option B+ (July 2014–December 2014)	Option B+ Implementation (January 2015–December 2015)	Pre-UTT (January 2016–August 2016)	UTT Implementation (September 2016–August 2017)	Post-UTT (September 2017–March 2019)
	Coefficient (95% CI) *P* Value	Coefficient (95% CI) *P* Value	Coefficient (95% CI) *P* Value	Coefficient (95% CI) *P* Value	Coefficient (95% CI) *P* Value
Underlying time trend^a^	–1.5 (–7.8 to 4.9) *P* = .647	N/A	N/A	N/A	N/A
Level change^b^	N/A	52.7 (30.9 to 74.5) *P* < .001	N/A	124.2 (102.2 to 146.1) *P* < .001	N/A
Trend change^c^	N/A	–1.7 (–8.3 to 4.8) *P* = .603	6.3 (2.6 to 10.0) *P* = .001	–8.7 (–11.9 to –5.5) *P* < .001	6.6 (3.9 to 9.3) *P* < .001
Absolute trend^d^	–1.5 (–7.8 to 4.9) *P* = .647	–3.2 (–4.7 to –1.7) *P* < .001	3.1 (0.5 to 5.7) *P* = .021	–5.6 (–7.5 to –3.8) *P* < .001	1.0 (–0.3 to 2.2) *P* = .122

The regression model included inverse probability weights for availability of a within-window CD4 count, and a covariate for sex.

Abbreviations: CI, confidence interval; N/A, not applicable; UTT, universal test and treat.

^a^ The underlying time trend refers to the “baseline” trend of CD4 count at antiretroviral therapy (ART) initiation in the analysis. Based on our models, this refers to the time trend in CD4 count at ART initiation during the pre-option B+ period. Subsequent “absolute” time trends for each study period are calculated from this baseline, drawing on the modeled trend changes as described below.

^b^Level changes were modeled at immediate policy change (option B+ policy change and UTT policy change) but not at 12 months after implementation of the policy.

^c^Each trend change is the change in trend relative to the (absolute) trend in the time period immediately preceding it. Therefore, the trend change for option B+ implementation relative to the pre-Option B+ trend is –1.7 cells/µL, and the trend change in pre-UTT relative to Option B+ implementation is +6.3 cells/µL.

^d^Absolute trends were calculated using regression post-estimation commands (lincom in Stata). For example, the absolute trend for Option B+ implementation = pre-Option B+ time trend plus Option B+ implementation trend change; the absolute trend for pre-UTT = pre-Option B+ time trend plus Option B+ implementation trend change plus pre-UTT trend change.

The overall mean CD4 count was 324.9 cells/μL (95% CI, 319.3 to 330.4) during Option B+ implementation (January 2015 to December 2015) and 317.1 cells/μL (95% CI, 308.6 to 325.6) pre-UTT (January 2016 to August 2016). Mean CD4 counts among women and men are presented in Supplementary Table 1.

#### September 2016 to March 2019 (UTT implementation and post-UTT periods)

Immediately after the policy switch to UTT in September 2016, there was a marked increase in mean CD4 count at ART initiation of 124.2 cells/μL (95% CI, 102.2 to 146.1). However, there was a downward absolute trend during the UTT implementation period (September 2016 to August 2017) of 5.6 cells/μL per month (95% CI, –7.5 to –3.8). The CD4 trend stabilized post-UTT (Table 1).

The overall mean CD4 count was 421.0 cells/μL (95% CI, 413.0 to 429.0) during UTT implementation (September 2016 to August 2017) and 389.5 cells/μL (95% CI, 381.8 to 397.1) post-UTT (September 2017 to March 2019). Mean CD4 counts among women and men are presented in Supplementary Table 1. Men initiated ART at lower CD4 counts than women after adjusting for policy changes and trend changes (–118.2 cells/μL, 95% CI, –125.5 to –111.0; Figure 4).

## DISCUSSION

We found that mean CD4 count at ART initiation significantly increased immediately after the policy change to UTT. However, the longer-term effect of UTT on mean CD4 count at ART initiation was modest. Following the initial UTT policy rollout, mean CD4 count at ART initiation trended downward before stabilizing at approximately 70 cells/μL above pre-UTT baseline CD4 counts. Women consistently initiated ART at higher CD4 counts than men. The proportion of individuals without baseline CD4 counts increased over time, particularly after the policy change to UTT. A large proportion of individuals had advanced HIV at ART initiation despite the eligibility expansion.

The relative proportions of individuals starting ART at lower vs higher CD4 counts influence our observed mean CD4 trends. If the majority of ART initiators had CD4 counts close to or above 500 cells/μL, we would expect the mean CD4 count after September 2016 to remain high and increase over time. Conversely, if lower CD4 count initiators remained the majority, clearing the backlog of previously ineligible individuals would result in a transient increase in mean CD4 count before approaching pre-UTT levels, as we observed. However, the more stable mean CD4 count after September 2017 of about 70 cells/μL above pre-UTT levels attests to an overall medium-term benefit of UTT on earlier ART initiation.

Several health service factors may explain these time trends and sex disparities in CD4 count at ART initiation after the UTT policy change. First, there may have been a transient expansion of HIV testing shortly after the UTT policy change at facilities, as illustrated by a process evaluation of a trial conducted at 7 clinics in the area [24]. Second, staffing and other resource shortages may have limited timely implementation of the policy due to limited training or competing clinical priorities [24, 25]. Moreover, Health workers may have selectively conducted baseline CD4 tests among individuals they perceived to be at risk of advanced HIV, thereby diluting the impact of UTT on CD4 count at ART initiation. Third, the historical policy focus on maternal and child health may have contributed to sex disparities in access to care [26, 27]. Inconvenient clinic operating hours or clinics being perceived as less “men friendly” [28] may have been additional factors. Men are more likely to start ART at later stages of HIV infection than women [29, 30]. Although higher CD4 counts among women may be attributed to earlier HIV diagnosis and treatment during pregnancy, a study in South Africa found that only 7% of women who initiated ART were pregnant [30].

If people living with HIV do not access care at high CD4 counts, they will not initiate ART at high CD4 counts. Although this study did not directly observe time of presentation, other studies have found that many individuals, particularly men, continue to present with low CD4 counts even in the UTT era [31, 32]. Linkage to care within 6 months of HIV diagnosis is poor in the AHRI catchment area, particularly among men [17]. Prevailing gender norms, including hegemonic masculinity, may partly explain limited HIV care-seeking due to perceptions of powerlessness [33].

Among the trials testing implementation of UTT prior to in-country policy change [34–38], median CD4 counts at ART initiation were 320–401 cells/μL and most participants were women [34, 39, 40]. Population-level reductions in HIV incidence were demonstrated in 2 UTT trials that included community-based HIV testing, facilitated linkage to care, and patient-centered clinical services [35, 37]. Other studies showed that UTT was associated with ART initiation within 30 days of enrollment in care in some countries but not others [41]. These findings further highlight various health system considerations that are extraneous to the new policy including health service delivery challenges (including clinic congestion and negative health worker attitudes) [42], individual patient readiness for ART [42], limited uptake of HIV testing due to low perceived risk or fear of stigma [43], and poor linkage to care [34]. Reassuringly, ART eligibility expansions do not appear to crowd out sicker patients despite increased demand for ART services in resource-poor settings [44, 45].

Our study adds to the emerging evidence for the impact of UTT on early ART initiation in sub-Saharan Africa. A key methodological strength of this quasiexperimental ITS analysis is the measurement of outcomes in a large group of individuals attending services in a rural subdistrict: assuming population characteristics do not change over time, major sources of confounding are unlikely [22] and enable strong policy conclusions to be drawn. By measuring CD4 count at ART initiation, our outcome also indirectly measures coverage of HIV testing and linkage to care.

Our study has some limitations. First, we sourced CD4 results from the national clinical database, TIER.Net, which relies on manual data entry from paper-based medical records; errors may have been introduced during data entry. It is also unknown whether the large number of missing CD4 counts reflects clinical process failure (from lack of sampling through to poor results turnaround) or gaps in TIER.Net record-keeping [46]. Second, the increasing proportion of missing CD4 values may have biased our results. However, we addressed this by assigning inverse probability weights in our regression model. Third, our findings may not be generalizable to other settings as factors that influence HIV testing, linkage to care, and ART initiation may differ. For instance, the Hawthorne phenomenon may have occurred at facilities in the subdistrict given the regular presence of AHRI research staff in several clinics [47]. Finally, our results may overestimate the impact of UTT on CD4 counts at initiation due to enhanced outreach activities in the AHRI catchment area.

Efforts to improve early ART initiation through enhanced HIV testing and linkage to care are critical, as are targeted interventions to boost male engagement with services. Interventions such as community-based multidisease screening [36], patient-centered and personalized services [36], financial incentives [48], and community-based ART initiation and monitoring for men [49] have shown success. While the scalability and sustainability of such interventions are unknown, the need for a holistic approach—such as integrating HIV services with noncommunicable disease services, addressing the wider determinants of health [50], reducing stigma, strengthening the health system including human and other resources, and improving health service quality—alongside more individualized interventions remains.

Although CD4 counts no longer influence the decision to start ART, they are crucial to inform opportunistic infection risk stratification, targeted clinical management, and advanced disease care packages [3, 4]. This is particularly important given that one-third of people with HIV in LMIC still initiate ART late [7, 12], and there is an increased risk of early mortality among those without a pretherapy CD4 count [51].

## CONCLUSIONS

Although UTT immediately increased earlier ART initiation, the longer-term effect was modest. An increasing proportion of ART initiators did not have a baseline CD4 count, and a large proportion were living with advanced HIV. Men started ART at lower CD4 counts than women. A multifaceted approach is required to improve service quality and address wider determinants of health. Further research is needed to ascertain the long-term effects of UTT including virologic suppression and HIV incidence.

## Supplementary Data

Supplementary materials are available at *Clinical Infectious Diseases* online. Consisting of data provided by the authors to benefit the reader, the posted materials are not copyedited and are the sole responsibility of the authors, so questions or comments should be addressed to the corresponding author.

ciab650_suppl_Supplementary_Materials

## Notes

***Author Contributions.*** Study conceptualization: M. J. S., T. B., J. B., H. M. Y., H. Y. K., F. T., C. I., M. S., and K. B. Data curation: H. M. Y. Formal analysis: H. M. Y., J. B., T. B., K. P., and A. J. Supervision: F. A. P., K. P., A. J., T. B., and J. B. Writing of the original draft: H. M. Y. Writing: review and editing: H. M. Y., J. B., H. Y. K., K. P., F. A. P., A. J., J. W. D. N., M. J. S., F. T., C. I., K. B., M. S., D. P., and T. B.

***Acknowledgments.*** The authors thank all study participants and members of the Demographic Health and Surveillance Community, the South African National Department of Health partners, and the Africa Health Research Institute (AHRI) Research Data Management team including Ms Thobeka Mngomezulu, Mr. Dickman Gareta and Dr. Kobus Herbst.

***Disclaimer.*** The funders had no role in study design, data collection and analysis, decision to publish, or preparation of the manuscript.

***Financial support.*** This research was funded in whole, or in part, by the Africa Health Research Institute (AHRI) Wellcome Strategic Core Award [201433/A/16/A]. The AHRI Population Intervention Platform is partially funded by the South African Population Research Infrastructure Network, South African Department of Science and Technology. H. M. Y. was supported by an Australian Government Research Training Program Scholarship, University of New South Wales (UNSW), Sydney, Australia. The Kirby Institute is funded by the Australian Government Department of Health and Ageing and is affiliated with the Faculty of Medicine, UNSW, Sydney. J. W. D. N. is supported by the Alexander von Humboldt Foundation. M. S. was supported by the National Institutes of Health (R01-MH114560-03) and the Bill & Melinda Gates Foundation (OPP1136774 and OPP1171600). T. B. is supported by the Alexander von Humboldt Professor Award, funded by the Federal Ministry of Education and Research; the Wellcome Trust; the National Institute of Child Health and Human Development of the National Institutes of Health (NIH; R01-HD084233); the National Institute on Ageing of the NIH (P01-AI112339); and the Fogarty International Centre of the NIH (D43-TW009775). M. J. S. receives funding and salary support from the NIH (R01-AI124718). J. B. receives funding and salary support from the National Institute of Child Health and Human Development of the NIH (R01-HD084233); the National Institute of Allergy and Infectious Diseases of the NIH (R01-AI152149); and the National Institute of Mental Health of the NIH (K01-MH105320).

***Potential conflicts of interest.*** F. A. P. reports grants and personal fees from Gilead Sciences, ViiV Healthcare, Merck Sharp & Dohme, and Janssen Pharmaceuticals outside the submitted work. C. I. reports grants and nonfinancial support for conference attendance from Gilead Sciences outside the submitted work. M. S. reports grants from the NIH, Bill & Melinda Gates Foundation, UK Medical Research Council, Wellcome Trust, and Unitaid outside the submitted work. All other authors report no potential conflicts.

***Availability of data and materials.*** Access to datasets can be provided by the AHRI research data management team. Requests can be made to the AHRI data repository via the AHRI website at https://www.ahri.org/research/.
