# Supplementary material for: CD4+ T-Cell Count at Antiretroviral Therapy Initiation in the “Treat-All” Era in Rural South Africa: An Interrupted Time Series Analysis
Source: Clin Infect Dis. 2021 Jul 26;74(8):1350–9. doi: 10.1093/cid/ciab650 (PMC9049265; doi:10.1093/cid/ciab650)
Supplement: ciab650_suppl_Supplementary_Materials [file ciab650_suppl_supplementary_materials.docx]

**Supplementary File.**

**CD4+ T-cell count at antiretroviral therapy initiation in the “treat all” era in rural South Africa: an interrupted time series analysis**

This file contains the following information:

- Additional Methods
- Table S1
- Table S2

**Additional information on study setting including clinic size**

The Africa Health Research Institute (AHRI) has operated a longitudinal population health and demographic surveillance system (HDSS) since 2003 in Hlabisa sub-district [1]. Individuals registered on the national antiretroviral therapy (ART) programme database, TIER.Net, are retrospectively linked to the AHRI HDSS. However, we included all individuals aged ≥16 years registered on TIER.Net in the sub-district in our analysis, rather than only those who were linked to the HDSS. Since January 2017, the AHRI HDSS routinely offers point-of-care home-based HIV testing and counselling, and active linkage-to-care support with text message reminders and/or telephone calls up to 4 weeks after the HIV diagnosis [2]. Additionally, AHRI research nurses and research assistants are based at 11 of the 17 clinics to facilitate linkage to care and ART initiation. Research assistants register clinic attendance on an AHRI database (ClinicLink), and research nurses support Department of Health (DoH) nursing staff to initiate ART under supervision of the clinic operational manager.

Clinicians and researchers of AHRI supported rollout of ART in the sub-district when ART first became available in the public sector, via the Hlabisa HIV Treatment and Care programme [3]. The HIV care programme was formally handed over to local DoH staff in 2013. Based on this experience of working with local services, and a recent process evaluation of continuous quality improvement (CQI) at seven primary care clinics in the sub-district [4], most of the 17 nurse-led primary care clinics in Hlabisa sub-district, are in small single-storey buildings with limited space and limited staff. Resources are also limited, albeit to varying degrees in each clinic [4]. A key difference between these clinics is the number of patients seeking care (patient workload), a reflection of the local community size and accessibility by public transport. We therefore defined clinic size by ART patient workload over 12 months: small, ≤175 patients; medium, 176−374 patients; large, ≥375 patients.

**Table S1. Number of women and men initiating ART by CD4-eligibility period and time period**

| **Variable** | **Women** | | | | | **Men** | | | | | | | | **Overall** | | | | | | | |
| --- | --- | --- | --- | --- | --- | --- | --- | --- | --- | --- | --- | --- | --- | --- | --- | --- | --- | --- | --- | --- | --- |
|  | **Pre-Option B+** | **Option B+** | | **UTT** | | **Pre-Option B+** | **Option B+** | | | **UTT** | | | | **Pre-Option B+** | **Option B+** | | | | **UTT** | | |
|  | *n*=1222^*^ | *n*=4245^*^ | | *n*=4757^*^ | | *n*=532^*^ | *n*=2031^*^ | | | *n*=2478^*^ | | | | *n*=1754^*^ | *n*=6276^*^ | | | | *n*=7235^*^ | | |
|  | Jul 2014 – Dec 2014 | Jan 2015 – Dec 2015 | Jan 2016 – Aug 2016 | Sep 2016 – Aug 2017 | Sep 2017 – Mar 2019 | Jul 2014 – Dec 2014 | Jan 2015 – Dec 2015 | | Jan 2016 – Aug 2016 | | Sep 2016 – Aug 2017 | | Sep 2017 – Mar 2019 | Jul 2014 – Dec 2014 | Jan 2015 – Dec 2015 | Jan 2016 – Aug 2016 | | | Sep 2016 – Aug 2017 | | Sep 2017 – Mar 2019 |
|  | *n*=1222 | *n*=2867 | *n*=1378 | *n*=2380 | *n*=2377 | *n*=532 | *n*=1316 | | *n*=715 | | *n*=1111 | | *n*=1367 | *n*=1754 | *n*=4183 | *n*=2093 | | | *n*=3491 | | *n*=3744 |
| **Age (IQR)** | | | | | | | | | | | | | | | | | | | | | |
|  | 28 | 29 | 28 | 29 | 28 | 35 | 34 | | 34 | | 33 | | 34 | 30 | 31 | 30 | | 30 | | 31 | |
|  | (23–34) | (24–36) | (23–35) | (23–36) | (23–35) | (29–42) | (29–42) | | (29–41) | | (29–41) | | (29–41) | (24–37) | (25–38) | (25–37) | | (25–38) | | (25–38) | |
| **Mean CD4, cells/μL^‡^ (95% CI)** | | | | | | | | | | | | | | | | | | | | | |
|  | 332.2 (321.7–342.7) | 361.6 (355.5–367.8) | 356.7 (347.6–365.7) | 458.0 (449.5–466.5) | 432.0 (423.7–440.3) | 214.0 (203.3–224.7) | 243.4 (236.2–250.6) | | 238.4 (229.0–247.9) | | 339.8 (330.7–348.9) | | 313.8 (305.3–322.3) | 296.9 (286.9–307.0) | 324.9 (319.3–330.4) | 317.1 (308.6–325.6) | | 421.0 (413.0–429.0) | | 389.5 (381.8–397.1) | |
| **CD4 category, n (%)^**^** | | | | | | | | | | | | | | | | | | | | | |
| ≤100 cells/μL | 134 | 263 | 144 | 173 | 189 | 139 | 283 | 154 | | 197 | | 259 | | 273 | 546 | | 298 | | 370 | 448 | |
|  | (11.0%) | (9.2%) | (10.4%) | (7.3%) | (8.0%) | (26.1%) | (21.5%) | (21.5%) | | (17.7%) | | (19.0%) | | (15.6%) | (13.1%) | | (14.2%) | | (10.6%) | (12.0%) | |
| 101- 200 cells/μL | 178 | 339 | 205 | 223 | 258 | 133 | 241 | 173 | | 191 | | 270 | | 311 | 580 | | 378 | | 414 | 528 | |
|  | (14.6%) | (11.8%) | (14.9%) | (9.4%) | (10.9%) | (25.0%) | (18.3%) | (24.2%) | | (17.2%) | | (19.8%) | | (17.7%) | (13.9%) | | (18.1%) | | (11.9%) | (14.1%) | |
| 201-350 cells/μL | 496 | 780 | 368 | 492 | 561 | 203 | 395 | 210 | | 278 | | 350 | | 699 | 1175 | | 578 | | 770 | 911 | |
|  | (40.6%) | (27.2%) | (26.7%) | (20.7%) | (23.6%) | (38.2%) | (30.0%) | (29.4%) | | (25.0%) | | (25.6%) | | (39.8%) | (28.1%) | | (27.6%) | | (22.1%) | (24.3%) | |
| 351-500 cells/μL | 200 | 1052 | 430 | 532 | 510 | 42 | 339 | 141 | | 197 | | 250 | | 242 | 1391 | | 571 | | 729 | 760 | |
|  | (16.4%) | (36.7%) | (31.2%) | (22.4%) | (21.5%) | (7.9%) | (25.8%) | (19.7%) | | (17.7%) | | (18.3%) | | (13.8%) | (33.2%) | | (27.3%) | | (20.9%) | (20.3%) | |
| >500 cells/μL | 214 | 433 | 231 | 960 | 859 | 15 | 58 | 37 | | 248 | | 238 | | 229 | 491 | | 268 | | 1208 | 1097 | |
|  | (17.5%) | (15.1%) | (16.8%) | (40.3%) | (36.1%) | (2.8%) | (4.4%) | (5.2%) | | (22.3%) | | (17.4%) | | (13.1%) | (11.7%) | | (12.8%) | | (34.6%) | (29.3%) | |

^*^ sample sizes presented are for individuals with a CD4 count available within window

^**^ percentages may not add up to 100% due to rounding

**^‡^** Generated using regression (interrupted time series) post-estimation commands.

The distribution of missing CD4 counts over time is presented in Figure 3.

**Table S2. Proportion without a CD4 within window among all ART initiators, by sex and clinic size at ART initiation**

| **Variable** | | **Women** | | | | **Men** | | | |
| --- | --- | --- | --- | --- | --- | --- | --- | --- | --- |
| **Clinic size^‡^, number initiating ART** | | Small | Medium | Large | Missing | Small | Medium | Large | Missing |
|  |  | *n*=2040 | *n*=5131 | *n*=7120 | *n*=7 | *n*=827 | *n*=2362 | *n*=3110 | *n*=2 |
| **Total with no CD4 within window, n (%)*** | | 218 (10.7%) | 1101 (21.5%) | 2752 (38.6%) | 3 | 67 (8.1%) | 347 (14.7%) | 845 (27.2%) | 1 |
| **Age category, n (%)*** | | | | | | | | | |
|  | 16-25 years | 99 (4.9%) | 526 (10.3%) | 1099 (15.4%) | 1 | 6 (0.7%) | 41 (1.7%) | 81 (2.6%) | 0 |
|  | 26-35 years | 87 (4.3%) | 383 (7.5%) | 1150 (16.2%) | 2 | 28 (3.4%) | 169 (7.2%) | 396 (12.7%) | 0 |
|  | 36+ years | 32 (1.6%) | 192 (3.7%) | 503 (7.1%) | 0 | 33 (4.0%) | 137 (5.8%) | 368 (11.8%) | 1 |

* Percentages may not add up to 100% due to rounding

**^‡^** Clinic size was determined by patient workload

**References**

1. Tanser F, Hosegood V, Barnighausen T, et al. Cohort Profile: Africa Centre Demographic Information System (ACDIS) and population-based HIV survey. Int J Epidemiol. **2008**;37:956-62.

2. Baisley KJ, Seeley J, Siedner MJ, et al. Findings from home-based HIV testing and facilitated linkage after scale-up of test and treat in rural South Africa: young people still missing. HIV Med. **2019**;20:704-8.

3. Houlihan CF, Bland RM, Mutevedzi PC, et al. Cohort profile: Hlabisa HIV treatment and care programme. Int J Epidemiol. **2011**;40:318-26.

4. Yapa HM, Dhlomo-Mphatswe W, Moshabela M, et al. A continuous quality improvement intervention to improve antenatal HIV care testing in rural South Africa: evaluation of implementation in a real-world setting. Int J Health Policy Manag. **2020** Oct 27. doi: 10.34172/ijhpm.2020.178
